# Endobronchial ultrasound: a minimally invasive technology to assist diagnosis of thoracic diseases

**DOI:** 10.31744/einstein_journal/2019MD4921

**Published:** 2019-08-30

**Authors:** Altair da Silva Costa, Addy Lidvina Mejia Palomino, Iunis Suzuki, Paulo Rogerio Scordamaglio, Marcelo Gervilla Gregorio, Marcia Jacomelli

**Affiliations:** 1 Hospital Israelita Albert Einstein, São Paulo, SP, Brazil.

**Keywords:** Mediastinum, Biopsy, fine-needle, Endoscopic ultrasound-guided fine needle aspiration/methods, Ultrasonography, interventional, Lymph nodes, Bronchoscopy

## Abstract

The endobronchial ultrasound is a minimally invasive technique that simultaneously associates ultrasound and bronchoscopy, to visualize lung nodule or masses, airway wall, and structures adjacent to the tracheobronchial tree. Endobronchial ultrasound has been incorporated into clinical practice all over the world because of its low risk and high diagnostic yield in neoplastic and non-neoplastic disease.

## INTRODUCTION

Since it was introduced by Hurter and Hanrath, in 1992, endobronchial ultrasound (EBUS) has been a useful technique that allows the bronchoscopist to see beyond the airways, including airway wall, lung and mediastinum. Endobronchial ultrasound is performed during bronchoscopy and permits sample collection for different thoracic disease. There are two types of EBUS: convex-probe EBUS (CP-EBUS) and radial-probe (RP-EBUS). These technologies will be discussed in the following topics.

### Convex endobronchial ultrasound

The currently available CP-EBUS is a dedicated ultrasound equipment, placed on the tip of a flexible bronchoscope to obtain images of the airways by direct contact of the probe with the tracheobronchial wall. The equipment has a working channel, Doppler function and a dedicated needle to perform a safe transbronchial needle aspiration, with ultrasound images, in real-time. The procedure is called EBUS transbronchial needle aspiration (EBUS-TBNA). All lesions near or in contact with the tracheobronchial tree can be accessed by EBUS-TBNA. In this way, the most frequent indications for EBUS-TBNA are diagnosis of mediastinal and hilar lesions of any etiology, mediastinal staging and restaging of lung cancer, staging of extra-thoracic cancer.^1^

The needle size varies from 19 to 25G, for histological and cytological, respectively. The needles available in Brazil are for cytological sampling only (21, 22 and 25G).

**Figure 1:**
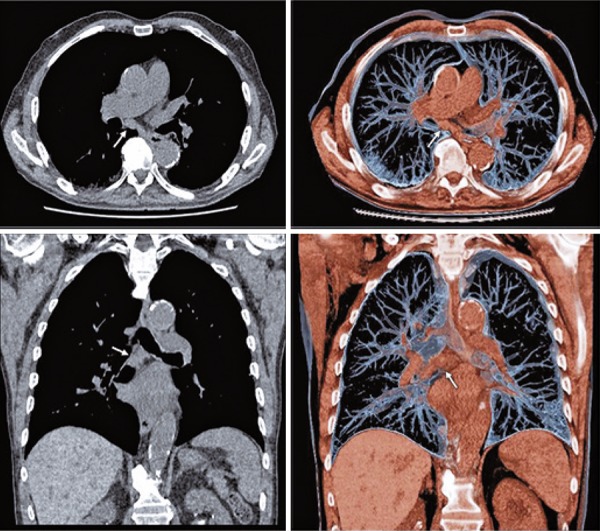
Chest tomography and subcarinal lymph node (station 7 - arrow)

**Figure 2:**
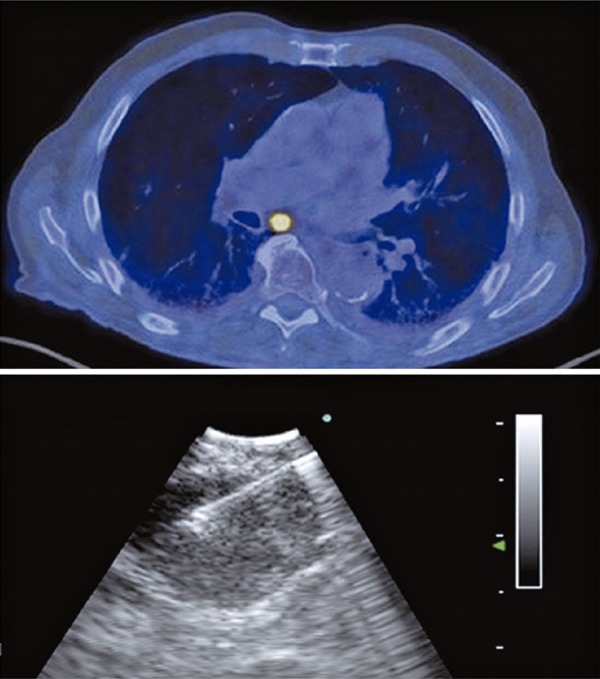
Positron emission tomography (SUV 12.6) and endobronchial ultrasound. Transbronchial needle aspiration with real-time lymph node puncture

Especially for mediastinal staging of lung cancer, EBUS-TBNA requires a systematic assessment. In this context, in 2009 the International Association for the Study of Lung Cancer (IASLC)^2^ established a mediastinal map and the lymph node stations were numbered from 1 to 14. The EBUS-TBNA can assess the stations 2, 4, 7, 10, 11 and 12. The assessment of each lymph node station depends on clinical indication, computed tomography (CT) or positron emission tomography CT (PET-CT) findings, and etiology of the tumor. For example, during mediastinal staging of lung cancer, it is mandatory to start from N3 station (from contralateral or mediastinal hilum), followed by N2 (mediastinal or ipsilateral subcarinal lymph node) and N1 nodes (ipsilateral hilum or lobar) at the end of exam. As we have a large number of lymph node stations to be evaluated, it is necessary to choose the suspected lymph node to be sampled by EBUS-TBNA according to ultrasonographic aspects. Malignant nodes tend to be round, with well-defined margin, heterogeneous, without central hilar structure. Furthermore, it is important to add this information to tomographic or PET-CT analysis. On figure 1 , there is an example of a male patient with lung cancer (adenocarcinoma) and a chest tomography with a large lymph node at station 7. The respective PET-CT showed a SUV of 12.6 and EBUS-TBNA was performed ( Figure 2 ).

Other important factor during EBUS-TBNA is the rapid on-site evaluation (ROSE) by a pathologist, to assess the representativeness of the sample and conduct the detailed specific analysis.^3^ The remaining material is placed in formalin for cellblock analysis. Molecular analysis for cancer can be carried out in EBUS-TBNA samples.^3 - 5^ If non-cancer is suspected during ROSE, the sample can be sent to microbiological analysis, flow cytometry or polymerase chain reaction (PCR). The sensitivity of EBUS-TBNA ranges from 84 to 96%.^4 - 7^

### Radial-probe endobronchial ultrasound

Radial-probe EBUS is performed using a 20mHz flexible delicate probe (UM-3R, Olympus Medical Systems Corp., Tokyo, Japan), which is inserted through the working channel of the conventional bronchoscope towards to the target pulmonary lesion. It allows a 360^o^surrounding view of the parenchyma. It also helps to identify the correct bronchus of the lesion, based on echogenicity differences between bronchus, lesion and normal tissue.^5 , 8^ In this way, the development of RP-EBUS has emerged to improve bronchoscopy diagnostic sensitivity and accuracy for pulmonary nodules and masses.^8^

**Figure 3:**
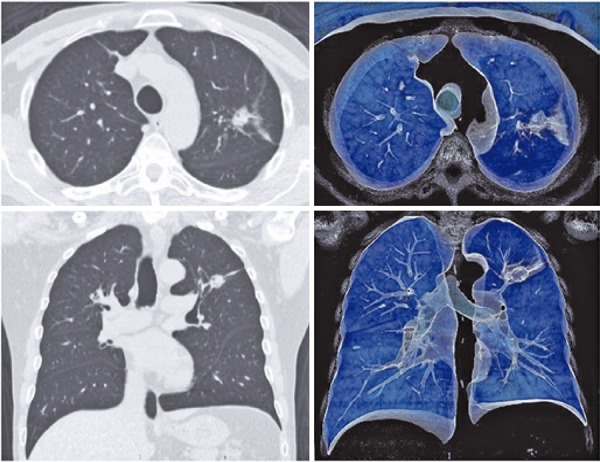
Upper left lobe nodule

**Figure 4:**
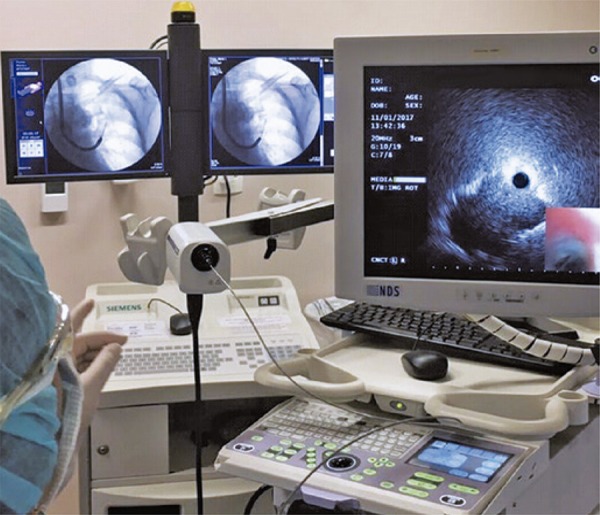
Bronchoscopy with radial endobronchial ultrasound and fluoroscopy for better visualization and performance of transbronchial biopsy of the pulmonary nodule in the upper left lung lobe

**Figure 5:**
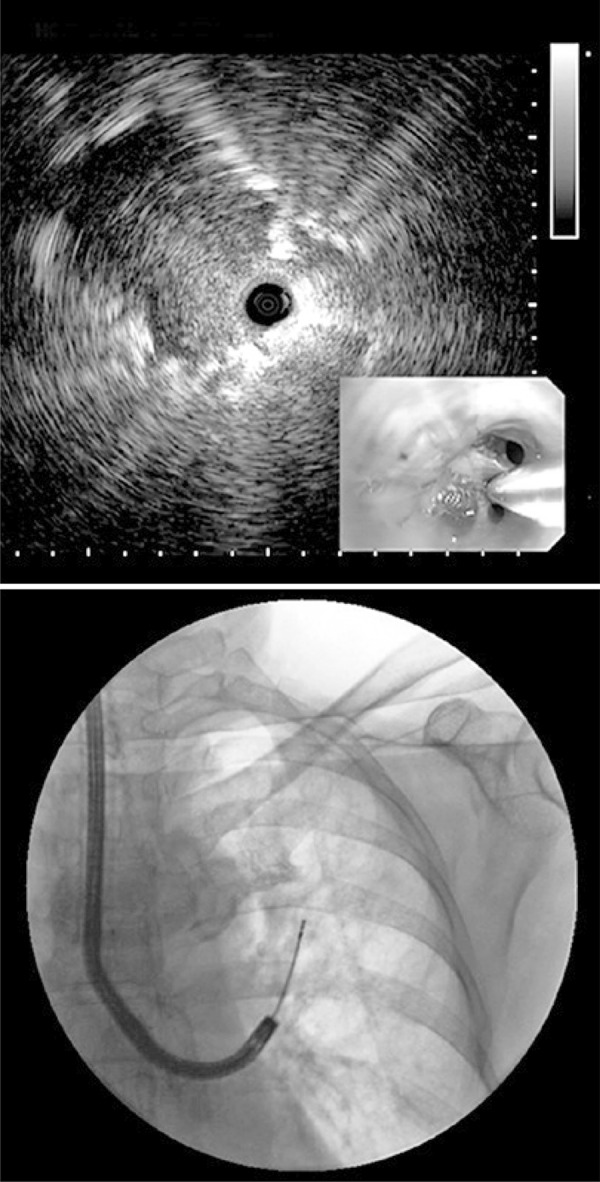
Radial endobronchial ultrasound and transbronchial biopsy guided by fluoroscopy

Radial-probe EBUS should be performed under fluoroscopy guidance to help evaluating the target lesion. Cytologic examination and transbronchial biopsies (brush and peripheral transbronchial needle aspiration) can be performed during the procedure, and the samples can be sent for microbiological, cytological and histological analysis, depending on clinical, radiological and cytological evaluation.^8 , 9^ For instance, figure 3 shows an upper left lobe nodule in a 72-year-old female smoker patient. We performed a bronchoscopy with radial EBUS and fluoroscopy ( Figures 4 and 5 ). The final diagnosis was lung adenocarcinoma.

Some factors, such as nodule size and capacity to visualize and locate the probe inside the lesion may affect the diagnostic yield of RP-EBUS. Our preliminary experience with RP-EBUS in Brazil showed good sensitivity for both nodules and masses (74.1 and 92%, respectively).^8^

EBUS-TBNA and RP-EBUS are safe procedures with low complication rates. The most common complication in EBUS-TBNA is damage to equipment during needle manipulation. Other complications like bleeding and infectious are rare. In RP-EBUS the complications are pneumothorax and bleeding, ranging from 1 a 4% and 3 a 5%, respectively, in most series.^9 , 10^

Table 1 displays the summary of characteristics of RP-EBUS and EBUS-TBNA.

**Table 1:** Summary of characteristics of radial-probe endobronchial ultrasound and endobronchial ultrasound-transbronchial needle aspiration

Topics	RP-EBUS	EBUS-TBNA
Characteristics	Conventional bronchoscopy with flexible probe	Dedicated bronchoscope with EBUS in the tip
	No doppler function	Doppler function
Target	Peripheral lung lesions (nodules and masses)	Mediastinal and hilar lesions (staging and restaging lung cancer, other neoplasms, inflammatory and infectious lesions)
Sensitivity	70 to 92%, depending on the characteristics of the lesion	84-96%
Associated techniques	Fluoroscopy, guide sheath	TBNA
	TBLB, TBNA, BAL	ROSE
	ROSE	
Complications	Pneumothorax (1-4%)	Overall (1.4%)
	Bleeding (3-5%)	Perforation by bronchoscope working channel

RP: radial-probe; EBUS: endobronchial ultrasound; TBNA: transbronchial needle aspiration; TBLB: transbronchial lung biopsy; BAL: bronchoalveolar lavage; ROSE: rapid on-site evaluation.

## CONCLUSION

Radial and convex endobronchial ultrasound is a safe procedure, with low complication rate and good sensitivity for both nodules and masses.
