# Supplementary material for: Endobronchial ultrasound: a minimally invasive technology to assist diagnosis of thoracic diseases
Source: Einstein (Sao Paulo). 2019 Aug 30;17(3):eMD4921. doi: 10.31744/einstein_journal/2019MD4921 (PMC6718194; doi:10.31744/einstein_journal/2019MD4921)
Supplement: Translation

# Translation: Ultrassonografia endobrônquica: tecnologia minimamente invasiva para auxiliar no diagnóstico de doenças torácicas

**Authors:** Altair da Silva Costa, Addy Lidvina Mejia Palomino, Iunis Suzuki, Paulo Rogerio Scordamaglio, Marcelo Gervilla Gregorio, Marcia Jacomelli

**Affiliations:** 1 Brasil Hospital Israelita Albert Einstein, São Paulo, SP, Brasil.

**Keywords:** Mediastino, Biópsia por agulha fina, Aspiração por agulha fina guiada por ultrassom endoscópico/métodos, Ultrassonografia de intervenção, Linfonodos, Broncoscopia

## Abstract

A ultrassonografia endobrônquica é uma técnica minimamente invasiva que associa simultaneamente broncoscopia à ultrassonografia, com a finalidade de visualizar nódulos ou massas pulmonares, paredes das vias aéreas, e estruturas ao redor de toda a árvore traqueobrônquica. A ultrassonografia endobrônquica foi incorporada à prática clínica em todo o mundo devido a seu baixo risco e elevado rendimento diagnóstico em doenças neoplásicas e não neoplásicas.

## INTRODUÇÃO

Desde sua introdução por Hurter e Hanrath, em 1992, a ultrassonografia endobrônquica (EBUS - *endobronchial ultrasound* ) tem se mostrado técnica útil, que permite ao operador visualizar além das vias aéreas, incluindo a parede das vias, o pulmão e o mediastino. A EBUS é realizada durante a broncoscopia e permite a coleta de amostras para diversas doenças torácicas. Há dois tipos de EBUS: a EBUS com transdutor convexo (CP-EBUS - *convex-probe* EBUS) e com transdutor radial (RP-EBUS - *radial-probe* EBUS). Essas tecnologias são discutidas nos tópicos a seguir.

### Ultrassonografia endobrônquica convexa

A CP-EBUS disponível atualmente usa um equipamento de ultrassonografia dedicado, colocado na ponta de um broncoscópio flexível, para obter imagens da região das vias aéreas, por contato direto do transdutor com a parede traqueobrônquica. O equipamento tem canal de trabalho, função Doppler e agulha dedicada para a realização segura de punção aspirativa transbrônquica, com imagens de ultrassonografia, em tempo real. O procedimento é conhecido como punção aspirativa transbrônquica guiada por EBUS (EBUS-TBNA). Todas as lesões próximas ou em contato com a árvore traqueobrônquica podem ser acessadas por EBUS-TBNA. Por isso, as indicações mais frequentes para EBUS-TBNA são diagnóstico de lesões mediastinais e hilares de qualquer etiologia, estadiamento mediastinal e restadiamento de câncer de pulmão, e estadiamento de câncer extratorácico.^1^

O tamanho da agulha varia entre calibre 19 e 25G, para análise histológica e citológica, respectivamente. As agulhas disponíveis no Brasil servem apenas para coleta de amostras citológicas (calibres 21, 22 e 25G).

Para o estadiamento mediastinal de câncer de pulmão, em particular, a EBUS-TBNA precisa incluir uma avaliação sistemática. Nesse contexto, em 2009, a *International Association for the Study of Lung Cancer* (IASLC)^2^ estabeleceu um mapa mediastinal, e as cadeias de linfonodos foram numeradas de 1 a 14. A EBUS-TBNA consegue acessar as cadeias 2, 4, 7, 10, 11 e 12. A avaliação de cada cadeia de linfonodos depende da indicação clínica, dos resultados da tomografia computadorizada (TC) ou da TC por emissão de pósitrons (PET-TC), e da etiologia do tumor. Por exemplo, durante o estadiamento mediastinal do câncer de pulmão, é obrigatório começar pela cadeia N3 (a partir do hilo contralateral ou mediastinal), seguida de N2 (linfonodo mediastinal ou subcarinal ipsilateral) e N1 (hilo ipsilateral ou lobar) no fim do exame. Como é grande o número de cadeias de linfonodos para avaliar, é necessário escolher o linfonodo suspeito do qual deve ser coletada a amostra para EBUS-TBNA, de acordo com aspectos ultrassonográficos. Linfonodos malignos tendem a ser arredondados, com margens bem definidas, heterogêneos e com estrutura hilar central. Além disso, é importante acrescentar essas informações à análise por tomografia ou PET-CT. A figura 1 mostra o exemplo de um paciente do sexo masculino, com câncer de pulmão (adenocarcinoma) e tomografia de tórax com linfonodo aumentado na cadeia 7. O PET-CT correspondente mostrou SUV de 12,6 e foi realizada EBUS-TBNA ( Figura 2 ).

**Figura 1:** Tomografia de tórax com linfonodo subcarinal (cadeia 7)

**Figura 2:** Tomografia de tórax por emissão de pósitrons (SUV 12.6) e punção transbrônquica do linfonodo, guiada por ultrassonografia em tempo real

Um outro fator importante durante a EBUS-TBNA é a avaliação rápida on-site (ROSE) por um patologista, para avaliar a representatividade da amostra e conduzir uma análise específica mais aprofundada.^3^ O material restante é colocado em formol para análise de blocos de células. É possível realizar análise molecular para câncer em amostras de EBUS-TBNA.^3 - 5^ Caso haja outra suspeita que não seja câncer durante a ROSE, a amostra pode ser enviada para análise microbiológica, citometria de fluxo ou reação em cadeia da polimerase (PCR). A sensibilidade da EBUS-TBNA varia de 84 a 96%.^4 - 7^

### Ultrassonografia endobrônquica com transdutor radial

A EBUS com RP-EBUS usa um transdutor delicado e flexível, de 20mHz (UM-3R, Olympus Medical Systems Corp., Tóquio, Japão), que é inserido pelo canal de trabalho de um broncoscópio convencional, em direção à lesão pulmonar a ser investigada. A técnica permite uma visão de 360^o^da região e ajuda a identificar o brônquio da lesão, com base nas diferenças de ecogenicidade entre este e o parênquima normal.^5 , 8^ Dessa forma, a RP-EBUS surgiu para melhorar a sensibilidade e a exatidão diagnóstica da broncoscopia na investigação de nódulos e massas pulmonares.^8^

A EBUS com RP-EBUS deve ser realizada sob fluoroscopia para auxiliar na avaliação da lesão-alvo. É possível realizar exame citopatológico e biópsia transbrônquica (escovado e punção aspirativa transbrônquica) durante o procedimento, e as amostras podem ser enviadas para análise microbiológica, citológica e histológica, dependendo da avaliação clínica, radiológica e citológica.^8 , 9^ Por exemplo, a figura 3 mostra um nódulo no lobo superior esquerdo em paciente do sexo feminino, tabagista, 72 anos. Realizamos broncoscopia com EBUS radial e fluoroscopia ( Figuras 4 e 5 ). O diagnóstico final foi adenocarcinoma pulmonar.

**Figura 3:** Nódulo no lobo superior esquerdo

**Figura 4:** Broncoscopia com ultrassonografia endobrônquica radial e fluoroscopia para melhor visualização e coleta de biópsia transbrônquica do nódulo no lobo superior do pulmão esquerdo

**Figura 5:** Ultrassonografia endobrônquica radial e biópsia transbrônquca guiada por fluroscopia

Alguns fatores, como tamanho do nódulo, capacidade de visualização e localização do transdutor dentro da lesão, podem afetar o rendimento diagnóstico da RP-EBUS. Nossa experiência preliminar com RP-EBUS no Brasil mostrou boa sensibilidade para nódulos e massas (74,1 e 92%, respectivamente).^8^

A EBUS-TBNA e a RP-EBUS são procedimentos seguros, com baixos índices de complicações. A complicação mais frequente na EBUS-TBNA corresponde aos danos aos equipamentos durante a manipulação da agulha. Outras complicações, como sangramento e infecções, são raras. Na RP-EBUS, as complicações são pneumotórax e sangramento, que variam de 1 a 4% e 3 a 5%, respectivamente, na maioria das casuísticas.^9 , 10^

A tabela 1 mostra o resumo de características da RP-EBUS e da EBUS-TBNA.

**Tabela 1:** Resumo de características da ultrassonografia endobrônquica com transdutor radial e da punção aspirativa transbrônquica guiada por ultrassonografia endobrônquica

Tópicos	RP-EBUS	EBUS-TBNA
Características	Broncoscopia convencional com transdutor flexível	Broncoscópio dedicado com EBUS na ponta
	Sem função Doppler	Função Doppler
Alvo	Lesões pulmonares periféricas (nódulos e massas)	Lesões mediastinais e hilares (estadiamento e reestadiamento de câncer de pulmão, outras neoplasias, lesões inflamatórias e infecciosas)
Sensibilidade	70 a 92%, dependendo da característica da lesão	84-96%
Técnicas associadas	Fluoroscopia, bainha-guia	TBNA
	TBLB, TBNA, LBA	ROSE
	ROSE	
Complicações	Pneumotórax (1-4%)	Geral (1,4%)
	Sangramento (3-5%)	Perfuração pelo canal de trabalho do broncoscópio

RP: *radial-probe* ; EBUS: ultrassonografia endobrônquica; TBNA: punção aspirativa transbrônquica; TBLB: biópsia pulmonar transbrônquica; LBA: lavagem bronco-alveolar; ROSE: avaliação rápida on-site.

## CONCLUSÃO

A ultrassonografia endobrônquica dos tipos radial e convexa é um procedimento seguro, com baixas taxas de complicações e boa sensibilidade para nódulos e massas.
